# Ultrametric networks: a new tool for phylogenetic analysis

**DOI:** 10.1186/1748-7188-8-7

**Published:** 2013-03-05

**Authors:** Alberto Apostolico, Matteo Comin, Andres Dress, Laxmi Parida

**Affiliations:** 1 College of Computing, Georgia Institute of Technology, 801 Atlantic Drive, Atlanta, GA 30332, USA; 2 Department of Information Engineering, University of Padova, Via Gradenigo 6/A, 35131 Padova, Italy; 3 CAS‐MPG Partner Institute and Key Lab for Computational Biology, Shanghai Institutes for Biological Sciences, Chinese Academy of Sciences, and infinity, Bielefeld, Germany; 4 IBM T. J. Watson Research Center, Yorktown Heights, NY 10598, USA

**Keywords:** Phylogenetic network, Ultrametric distance, STR data analysis

## Abstract

**Background:**

The large majority of optimization problems related to the inference of distance‐based trees used in phylogenetic analysis and classification is known to be intractable. One noted exception is found within the realm of ultrametric distances. The introduction of ultrametric trees in phylogeny was inspired by a model of evolution driven by the postulate of a molecular clock, now dismissed, whereby phylogeny could be represented by a weighted tree in which the sum of the weights of the edges separating any given leaf from the root is the same for all leaves. Both, molecular clocks and rooted ultrametric trees, fell out of fashion as credible representations of evolutionary change. At the same time, ultrametric dendrograms have shown good potential for purposes of classification in so far as they have proven to provide good approximations for additive trees. Most of these approximations are still intractable, but the problem of finding the nearest ultrametric distance matrix to a given distance matrix with respect to the *L*_*∞*_ distance has been long known to be solvable in polynomial time, the solution being incarnated in any minimum spanning tree for the weighted graph subtending to the matrix.

**Results:**

This paper expands this *subdominant ultrametric* perspective by studying ultrametric *networks*, consisting of the collection of *all* edges involved in some minimum spanning tree. It is shown that, for a graph with *n* vertices, the construction of such a network can be carried out by a simple algorithm in optimal time *O*(*n*^2^) which is faster by a factor of *n* than the direct adaptation of the classical *O*(*n*^3^) paradigm by Warshall for computing the transitive closure of a graph. This algorithm, called UltraNet, will be shown to be easily adapted to compute relaxed networks and to support the introduction of artificial points to reduce the maximum distance between vertices in a pair. Finally, a few experiments will be discussed to demonstrate the applicability of subdominant ultrametric networks.

**Availability:**

http://www.dei.unipd.it/~ciompin/main/Ultranet/Ultranet.html

## Background

As is well known, most optimization problems related to the inference of distance‐based trees used in phylogenetic analysis and classification are intractable (see [1,2] for a pertinent discussion). One notable exception is found within the realm of ultrametric distances (cf. [3]). The introduction of such distances in phylogeny was inspired by a model of evolution, now largely abandoned, driven by the postulate of a molecular clock whereby the amount of phylogenetic change observable between any two extant species is directly related to the amount of time that elapsed since their last common ancestor roamed this planet, implying that phylogenetic distances could simply be represented by a weighted tree in which the sum of the weights of the edges separating any given leaf from the root is the same for all leaves.

Both molecular clocks and rooted ultrametric trees fell out of fashion as credible representations of evolutionary change. At the same time, a rooted dated tree is still the “object of desire” in taxonomy and Tree‐of‐Life research, and ultrametric dendrograms have shown good potential for purposes of classification in so far as they have proven to provide good approximations for additive trees. While finding the “best” such approximation is, in most cases, still intractable, the problem of finding an ultrametric distance matrix that is closest to a given distance matrix with respect to the *L*_*∞*_ distance has long been known to be solvable in polynomial time, its solution being incarnated in any minimum spanning tree for the weighted graph subtending to the matrix.

Applications of minimum spanning trees in connection with problems of population classification and genetics are as old as any other of their numerous applications. An application to taxonomic problems related to species interrelationship dates back to [4]. And as early as 1964, Edwards and Cavalli Sforza [5] used MSTs to approximate evolutionary trees reconstructed from gene frequencies in blood groups from fifteen contemporary human populations.

Most approximation problems arising in this context fall within the framework of the following

**Closest Metric Problem:***Given a set*M*of metrics C defined on a set V, an* |*V*|×|*V*|−*matrixM*, *and a distance function*$D:(M^{\prime },M^{\prime \prime })\to R_{\geq 0}$*defined on the set*$R^{\left|V|\times |V\right|}$*of all* |*V*|×|*V*|−*matrices*, *find a metric*C∈M*with minimum distance to M relative to D*.

The basic facts are summarized in Table 1.

**Table 1:** Basic facts for the closest metric problem

***C *∖ *D***_***M***_	***L***_***1***_	***L***_***2***_	***L***_***∞***_
Additive	NP‐Hard^+^	NP‐Hard	NP‐Hard^*^
Ultrametric	NP‐Hard^+^	NP‐Hard	P^§^

^+^no approximation is known;

^*^a 3‐approximation exists, due to Agarwal et al. [1];

^§^due to Gower and Ross [3] (see also ([6], p.158), ([7], p.134), [2,8]).

Subdominant ultrametrics have been traditionally applied to many problems of physics and optimization theory [9]‐[12]. More recently, implications of this theory in the analysis of financial markets, stock exchange, and evolutionary biology have attracted new interest in the topic.

Phylogenetic networks are increasingly featured in modeling of molecular evolution, as evidence of reticulate events such as hybridization, horizontal gene transfer and recombination becomes more prominent. Traditionally, the use of binary data and, in particular, the notion of splits gave rise to a number of alternative models. In the literature, several definitions of networks have been proposed to model parallel events. Popular examples are consensus networks [13], reticulate networks, recombination networks, median networks [14], Neighbour Nets [15], QNets [16] etc. In order to control the degree of connectivity of a network, each model optimizes an objective function; examples are Bayesian methods, maximum likelihood methods, and maximum parsimony [17,18], calculated that the number of equally parsimonious trees for a data set of just 56 haplotypes exceeded one billion. This estimate was computed through resort of the notion of Minimum Spanning Network. In a different context, they proposed a counting procedure based on the Prim’s algorithm that is analogous to the work presented in this paper.

Anther popular framework is the statistical parsimony analysis [19]. Hart and Sunday [20] found empirically that subnetworks, as implemented in the TCS program [21], coincided significantly with taxonomy names. The TCS program calculates the maximum number of mutational steps constituting a parsimonious connection between two haplotypes with the probability of 95%. Although Hart and Sunday’s [20] results suggest that statistical parsimony analysis could be used in practice to differentiate species, this methodology is not mathematically well‐founded.

In this paper, we extend the approach based on the construction of subdominant ultrametric trees by studying ultrametric *networks*, consisting of the collection of all edges involved in some minimum spanning tree. This can be viewed as a network of kinship between the extant sequences that embodies the *least‐resistant paths* in terms of *bottlenecks*, where a bottleneck is simply the worst possible transition between two intermediate states. We show that, for a graph with *n* vertices, the construction of such a network can be carried out by a simple algorithm in optimal time *O*(*n*^2^), which is faster by a factor of *n* than the more straightforward *O*(*n*^3^) closure performed by the classical Floyd‐Warshall paradigm. We show that our algorithm can easily be adapted to compute relaxed networks and to support the introduction of artificial points when it is desirable to reduce maximum distance between vertices. Finally, we discuss a few experiments demonstrating the applicability of this method.

## The ultrametric network

We study the following, rather abstract, conceptual frame work: We start with a finite set *V* representing the sequences and an arbitrary weighting

W:V2→R>0:{v,u}→W(v,u)

that associates a positive weight *W*(*v*,*u*) to every 2‐subset {v,u}∈V2 of *V* that we imagine to be deduced, in one way or the other, from the given sequences, and to represent, for every {v,u}∈V2, the observed *degree of dissimilarity* between *u* and *v*.

It is well known (cf. [3]) and easy to see (cf. [8] for a review) that there exists a unique largest *ultrametric* defined on *V* and denoted by, say, *W*^∗^ that is *dominated* by *W*, i.e., the (necessarily unique and symmetric) largest map from *V*×*V* into R for which 

(i) *W*^∗^(*v*,*v*)=0 and *W*^∗^(*v*,*u*)≤ max(*W*^∗^(*v*,*w*),*W*^∗^(*u*,*w*)) holds for all *u*,*v*,*w* in *V*, and

(ii) *W*^∗^(*v*,*u*)≤*W*(*v*,*u*) for all *u*,*v*∈*V*.

Actually, as the supremum

supD:V×V→R:(u,v)↦sup(D(u,v):D∈D)

of any collection D of ultrametrics defined on *V* that is bounded from above, is an ultrametric, too, and *W*^∗^ is just the supremum of the set,

D(W):={D:Dis an ultrametric onVthat is bounded from above byW},

*W*^∗^ must indeed be an ultrametric, called the *subdominant ultrametric* for *W*.

In this paper, we will study the *ultrametric networkG*(*V*|*W*) associated with *W*, i.e. the graph *G*(*V*|*W*):=(*V*, *E*(*V*|*W*)) with vertex set *V* and edge set E(V|W):={u,v}∈V2:W(u,v)=W∗(u,v).

It is easy to see that *E*(*V*|*W*) is actually the union of the edge sets of all minimum spanning trees with vertex set *V* relative to *W*, considered as a weighting of the complete graph *G*(*V*)=(*V*,*E*(*V*)) with vertex set *V* and edge set E(V):=V2.

Indeed, continuing with the notations and assumptions introduced above, *W*^∗^ can be constructed as follows: Put |*e*|:=*W*(*u*,*v*) for every *e*={*u*,*v*}∈*E*(*V*) and, given any path *P*=*v*_0_*v*_1_...*v*_*k*_ in *G*(*V*), define the *supportsupp*(*P*) of *P* by

$$\text{supp}(P):=\left\{\{v_{i-1},v_{i}\}:i=1,2,\ldots ,k\right\},$$

and the *bottleneckB*(*P*)=*B*(*P*|*W*) of *P* (with respect to *W*) by

$$B(P)=B(v_{0}v_{1}\mathrm{...}v_{k}):=\underset{i=1\mathrm{...k}}{\max}\{|e|:e\in \text{supp}(P)\}.$$

Then, given any two vertices *u*,*v*∈*V*, *W*^∗^(*u*,*v*) coincides with the *least‐resistance* bottleneck between *v* and *u*, i.e., we have

$$W^{*}(u,v)=\underset{\text{all paths}\text{P}\text{in}\;G(V)\text{from}u\text{to}v}{\min}B(P).$$

Any path for which this minimum is attained represents a *minimum‐bottleneck* path (for *u* and *v*). Clearly, *W*^∗^(*u*,*v*) is the lowest weight possible for the highest weight in any path leading from *u* to *v*. It can be computed by a straightforward adaptation of the Floyd‐Warshall algorithm in *O*(|*V*|^3^) time.

Remarkably, *E*⊆*E*(*V*|*W*) holds for every subset *E* of *E*(*V*) for which the graph (*V*,*E*) is connected and minimizes the sum $\left|E|:=\underset{e\in E}{\sum }|e\right|$, that is, for the edge set of any *minimum spanning treeT*=(*X*,*E*) for *W*. This follows from a result generally credited to [3], that we formalize as follows:

### Theorem 1

*With V and W as above, the edge set of every minimum spanning tree for W is contained inE*(*V*|*W*) *while, conversely, there exists, for any edgee*∈*E*(*V*|*W*), *a minimum spanning tree forWwhose edge set containse*. *In particular, the networkG*(*V*|*W*) *is always connected.*

### Proof

Indeed, given any such subset *E*⊆*E*(*V*) and any edge *e*={*u*,*v*}∈*E*, we may denote by *Π*(*e*)=*Π*_*E*_(*e*) the bi‐partition of *V* given by the (vertex sets of the) two connected components of the graph (*V*,*E*−{*e*}), and by *A*(*w*)=*A*_*E*_(*w*), for any *w*∈*V*, the unique component *A*(*w*)∈*Π*(*e*) with *w*∈*A*(*e*). Clearly, we have *Π*(*e*)={*A*(*u*),*A*(*v*)} for every edge *e*={*u*,*v*}∈*E*.

Now, assume that there exists some *e*={*u*,*v*}∈*E* with *e*∉*E*(*V*|*W*). Then, we could find some *P*=*v*_0_*v*_1_...*v*_*k*−1_*v*_*k*_ from *v*_0_:=*u* to *v*_*k*_:=*v* in *G*(*V*) with *B*(*P*)<|*e*|. Furthermore, as *A*(*v*_0_)=*A*(*u*)≠*A*(*v*)=*A*(*v*_*k*_) must hold, there must be some i∈{1,…,k} with *A*(*v*_*i*−1_)≠*A*(*v*_*i*_), eg the smallest *i* in {1,…,k} with *A*(*u*)≠*A*(*v*_*i*_). Consequently, exchanging the edge *e* with the edge *e*_*i*_:={*v*_*i*−1_,*v*_*i*_} in *E* would also give rise to a spanning tree for *G*(*V*), and we would have |*E*^′^|=|*E*|+|*e*_*i*_|−|*e*|<|*E*| in view of |*e*_*i*_|≤*B*(*P*)<|*e*|, thus contradicting our choice of *E*. So, *E*⊆*E*(*V*|*W*) must hold, as claimed.

To establish the converse, assume that *e*={*u*,*v*}∈*E*(*V*|*W*) is not contained in any minimum spanning tree. Then, given any such tree, let *P*=*v*_0_*v*_1_...*v*_*k*−1_*v*_*k*_ denote the unique path from *v*_0_:=*u* to *v*_*k*_:=*v* in that tree. Then, exchanging any edge *e*^′^ in the support of *P* with the edge *e* will produce a spanning tree for *G*(*V*) of larger weight, implying that |*e*^′^|<|*e*| must hold for every such edge *e*^′^ implying that also *B*(*P*)<|*e*| must hold. This, however, would clearly contradict our assumption *e*∈*E*(*V*|*W*). □

## Optimal computation of the ultrametric network

Clearly, given *V* and *W* as above, the ultrametric network can be produced in time *O*(|*V*|^3^) by a straightforward adaptation of the Floyd‐Warshall all‐pairs shortest‐path algorithm [22]. In view of Theorem 1, this network could be produced in time *O*(|*V*|^3^) also by first computing one MST by, say, Prim’s algorithm, and then computing *W*^∗^ using the paths in this tree. We present here an algorithm to compute the entire network in time *O*(|*V*|^2^). This is optimal since any algorithm must produce *Θ*(|*V*|^2^) values at the outset.

The main idea is that the computation can be cast within a control structure that is strongly reminiscent of Prim’s MST ‐ or Dijkstra’s single‐source shortest‐path algorithm (refer to, e.g., [22]): starting with an arbitrary vertex *r*, a subset $\bar{V}$ of *V* is progressively expanded by annexing, at each step, the one vertex *u* in $V-\bar{V}$ that is connected to $\bar{V}$ by an edge (*v*,*u*) that minimizes cost. As is well known, in Prim’s MST the cost to be minimized is the weight of the partial tree over the vertices in $\bar{V}$, whence the edge to be chosen is one of minimum weight. In Dijkstra’s algorithm, the cost to be minimized is the sum of weights on the arcs connecting *u* to *r*, whence the edge to be chosen is the one minimizing this sum. Note that in both cases there can be more than one vertex that minimizes the cost, however they will all produce the same global minimum. One important point of our algorithm is that (see Theorem 1) choosing (*v*,*u*) as in Prim’s MST computes the ultrametric distance not only between *u* and *r* but between *u* and any other vertex in $\bar{V}$. Moreover, it can be seen that the pairwise ultrametric distances between any pair of vertices in $\bar{V}$ are not affected by the introduction of *u* in this set. This last circumstance yields the speedup from *O*(|*V*|^3^) to *O*(|*V*|^2^).

The algorithm starts with the original weights *W*(*u*,*v*), computes the ultrametric *W*^∗^ and identifies the subset of edges that form the ultrametric network *Ē*. In the following pseudo‐code, *d*(*u*,*v*) is initialized to *∞* and then used to store consecutively refined estimates of the value of *W*^∗^(*u*,*v*), for any pair *u* and *v* of vertices. It will be seen that at the end *d*(*u*,*v*)=*W*^∗^(*u*,*v*)

### Lemma 1

*At each iteration, d coincides with*${\left(W{|}_{\bar{V}}\right)}^{*}$, *the subdominant ultrametric of the restriction*$W{|}_{\bar{V}}$*of W to*$\bar{V}$, *where the edge* {*v*^′^,*v*^″^}∈*Ēif and only if:*

• *d*(*v*^′^,*v*^″^)=*W*(*v*^′^,*v*^″^) and *d*(*v*^′^,*v*^″^)≤*max*(*W*(*v*^′^,*u*),*W*(*v*^″^,*u*)) holds for any two vertices *v*^′^,*v*^″^ in $\bar{V}$ and all *u*∈*Q*,

• $\text{key}[u]=W(\text{prec}[u],u)=\min(W(u,v):v\in \bar{V})$ holds for every $u\in Q=V-\bar{V}$.

**Figure 1:**
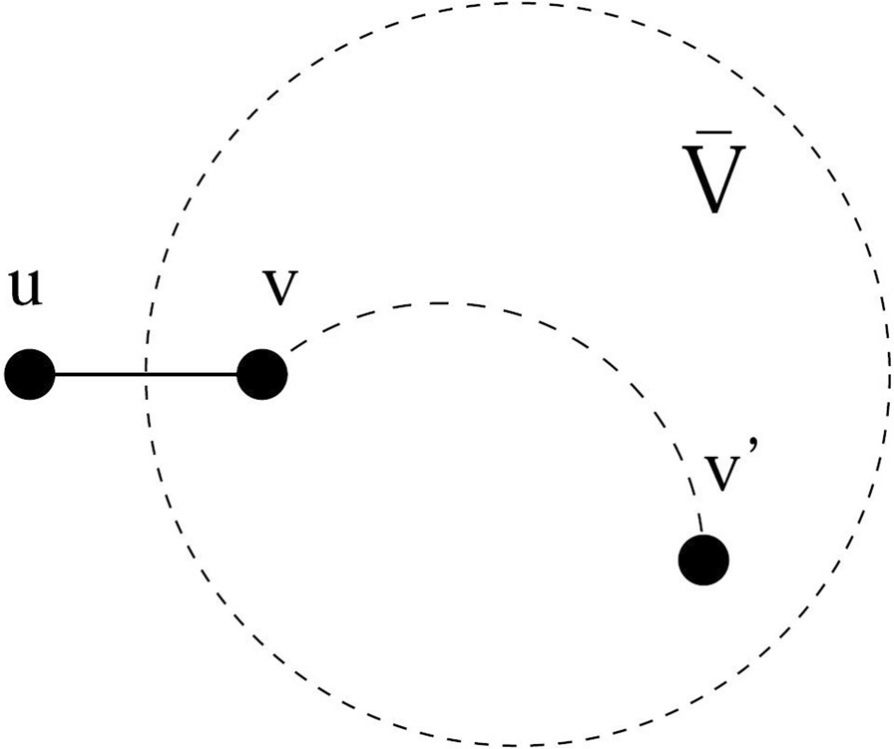
Illustrating the construction of the ultrametric network.

The proof follows easily from observing that $\bar{V}$ contains, at each recursive step, a connected subgraph that is part of a MST for V. Figure 1 shows the generic step of the algorithm. It is easy to check that the set *Ē* contains all edges of the ultrametric network. We now prove that the algorithm is also optimal.

### Lemma 2

*The algorithm constructs the subdominant ultrametric and its associated ultrametric network in optimal timeO*(|*V*|^2^).

### Proof

All the initialization steps, inclusive of the insertion of (|*V*|−1) initial values in the queue *Q* take time *O*(|*V*|^2^). Following this, each of the (|*V*|−1) iterations of the *while* loop contains two cascaded *for* cycles of *O*(|*V*|) elementary steps each. The first *for* computes the ultrametric network for the vertices in $\bar{V}$, whereas the second one updates the queue *Q*, which stores all vertices $v\notin \bar{V}$ according to the index *key*[*v*]. All the operations in each *for* take trivially constant time, except for the queue updates. If the queue is implemented as a Fibonacci heap, we can extract the minimum element in amortized *O*(log|*V*|) and update the queue in amortized *O*(1), when *key*[*v*] is decreased. There are (|*V*|−1)*extractmin* (at the beginning of every iteration of the *while*), which thus charge *O*(|*V*| log|*V*|) overall. There are *O*(|*V*|^2^) constant‐time updates throughout all the executions of the second *for* loop. Hence the total cost of the algorithm is *O*(|*V*|^2^). The subdominant ultrametric requires *Θ*(|*V*|^2^) entries, and an ultrametric network contains at most |*V*|^2^ edges, so that *O*(|*V*|^2^) time is optimal. □

## Ultrametric network relaxations

The algorithm of the preceding section lends itself naturally to variants that accommodate some tolerance in the ultrametric distance and relax the notion of ultrametric network. We outline here these two variants, respectively leading to *Δ*‐ultrametric networks and to the introduction of new artificial vertices.

### *Δ*‐Ultrametric extension

We define the *Δ‐ultrametric network* in which edges are inserted if their weights do not deviate more that a given threshold *Δ* from the corresponding ultrametric distance.

Formally the *Δ‐ultrametric network* consists of the graph *G*_*Δ*_(*V*|*W*)=(*V*, *E*_*Δ*_(*V*|*W*)) with vertex set *V* and edge set EΔ(V|W):={u,v}∈V2:W(u,v)≤W∗(u,v)+Δ, where *W*^∗^(*u*,*v*) is the standard ultrametric distance. Intuitively, the *Δ*‐ultrametric network is thus a relaxation of the ultrametric network, resulting in increased connectivity. More precisely the graph *G*_*Δ*_(*V*|*W*) coincides with the map min(*W*,*W*^∗^+*Δ*).

The *Δ*‐ultrametric network can be computed as a postprocessing by adding all such edges *E*_*Δ*_(*V*|*W*) to the *exact* ultrametric network. In summary at first we run the algorithm on the original weights *W*(*u*,*v*) to compute *W*^∗^. Then, we apply the postprocess that includes all edges (*u*,*v*) with weight *W*(*u*,*v*) that deviates at most *Δ* from the corresponding ultrametric value *W*^∗^(*u*,*v*). Similarly to the main algorithm, this postprocess takes optimal *O*(|*V*|^2^) time. Other alternatives relaxations can be explored, like the subdominant *Δ*‐ultrametrics for which analogous results can be established. The subdominant *Δ*‐ultrametrics relaxation will be addressed in a future paper.

### Artificial vertices

In applications such as phylogeny on biological data of extant species/individuals, the topology must account for missing data points. In other words, there is a need to reduce the distance between a pair of vertices by introducing a new artificial vertex in the network.

In the phylogeny construction problem, the given data points are the terminals and the artificial vertices correspond to missing (or ancestral) data points. The traditional Steiner tree problem [23] involves the minimization of the sum of the lengths of all edges used after introducing artificial vertices, as opposed to the sum of the pairwise distances of all the terminals. For different metrics the Steiner tree problem is known to be NP‐Hard [24]. Thus in our context, given the graph induced by the ultrametric *W*^∗^, the problem of introducing new artificial vertices that minimize the sum of the weights of all edges is still NP‐Hard.

In our case the input graph *G* is not just any graph, but it can be characterized as follow. Suppose we are given a (big) metric space R=(V,D) (could be the metric space consisting of the vertex set *R* of a connected weighted graph *G* with the “induced" metric, i.e., the largest metric *D* on *V* with *D*(*u*,*v*)≤*w*(*u*,*v*) for all edges *u*,*v* in *G*), and a finite subset *R* of *V*.

The solution to the Steiner tree problem is to find a connected graph G(R|V) with a vertex set containing *R* and contained in *V* and edge set E(R|V) such that $\underset{\left\{u,v\}\in E(R|V\right)}{\sum }D(u,v)$ is minimized. In case you just have a metric *D* on *V*, in our case *W*^∗^, the most natural choice for (*V*,*D*) is the tight span

$$T(D):=\{f\in R^{V}:f(v)=\sup(D(u,v)-f(u)):u\in V\}$$

of (*V*,*D*). It is natural in this case as any Steiner tree for *V* can be mapped into *T*(*D*) by a non‐expanding map that preserves the distances between the points in *V*[25]. We don’t know how to efficiently search for the best Steiner tree within *T*(*D*), also known as the optimal realization, without an exhaustive enumeration [26,27]. Instead starting from a given graph $G_{W^{*}}$ over *R* we look at the “neighborhood" of this seed in *T*(*D*). The input graph $G_{W^{*}}$ over *R* is the ultrametric network computed in the previous section and we are interested in the “neighborhood" of this network such that the sum of all edges is smaller and that the pairwise distances in *R* are preserved.

**Figure 2:**
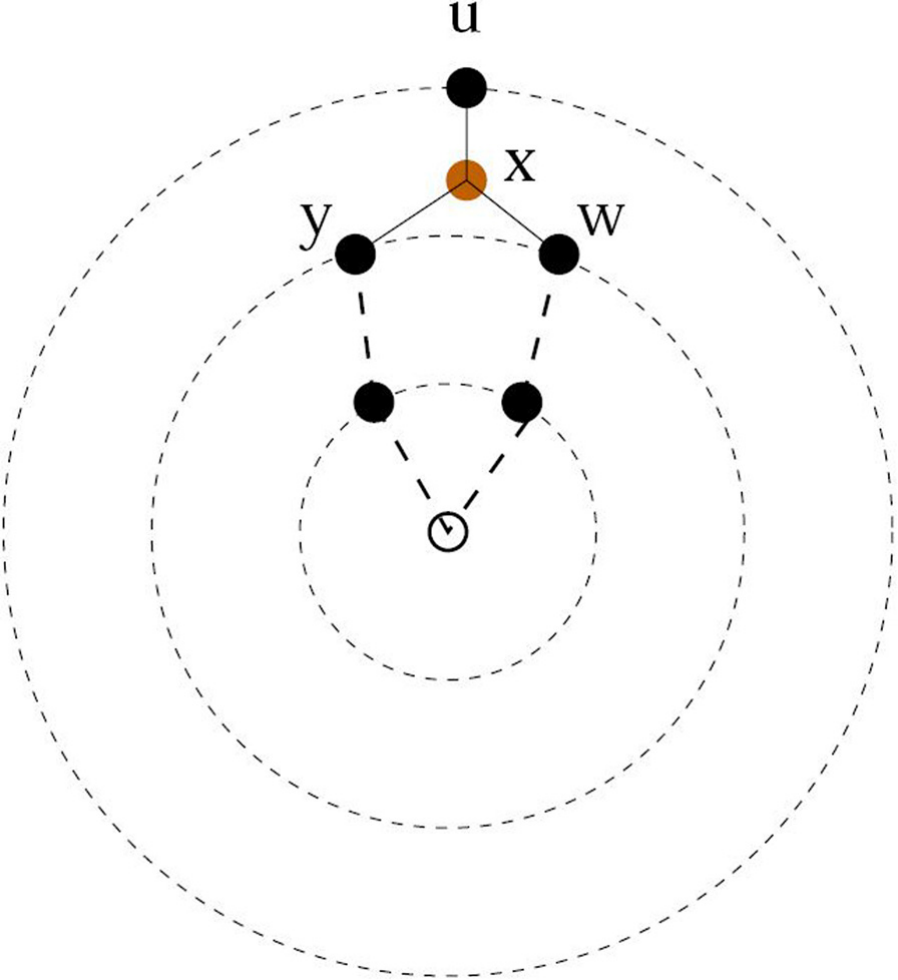
The creation of a new artificial vertex.

For every edge {*y*,*w*} in $G_{W^{*}}$ we search in its neighborhood by looking at the vertices directly connected to *y* and *w*. If there is some vertex *u* that is connected to *y* and *w* we explore the possibility to insert a new node *x* as the median of *y*, *w* and *u*. Clearly if we add the artificial vertex *x* and replace the edges that create a cycle (*u*,*w*), (*u*,*y*) and (*w*,*y*), with the edges (*u*,*x*), (*w*,*x*) and (*y*,*x*) (see Figure 2) this new configuration does not increase the contributions of the distances involving the three nodes.

To control the number of artificial vertices, the new vertex *x* is created only if the sum of pairwise distances of the triangle among *u*, *w* and *y* exceeds the threshold *Δ*. Note that if two triangles share an edge we need to select where to insert the new artificial vertex. A canonical order can be established by ranking all candidate triangles by the sum of pairwise distances. This ensures that, at least generically, the introduction of new artificial vertices is unique and does not depend on the input order.

## Experimental results

To conclude our presentation, we report two examples of inference of Human Y‐chromosome phylogeny from Short Tandem Repeats. This can be based on the study of Human migration and the associated relationships among different populations. In typical experiments, we are interested in constructing a network from the STRs information of various individuals and in comparing the results with known paths of migrations. An interesting example of such a phylogeny reconstruction can be found in [28], which discusses the significance of STRs data as markers for human evolution, but also highlights the difficulties that the analysis of this data derives from the lack of an appropriate methodology.

Using the same data of [28,29], we study migration histories within two different scenarios. In the first experiment, we analyze a very broad spectrum of populations: Africans, Europeans, Asians and Australians. In the second, we concentrate on Native Americans spanning North America (Navajo, Zuni, Sioux), Central America (Maya), and South America (Ticuna, Wichi, Toba, Chorote, Tehuelche, Susque, Humahuaqueño).

For both experiments the data available include a number of different STRs, specifically, 12 in the first experiment and 7 in second. The first step is to establish for all STRs a weighting scheme reflecting the different mutation rates. To this end, we use the three weights 1,2 and 4, and assign to each STR a weight proportional to its mutation rate.

**Figure 3:**
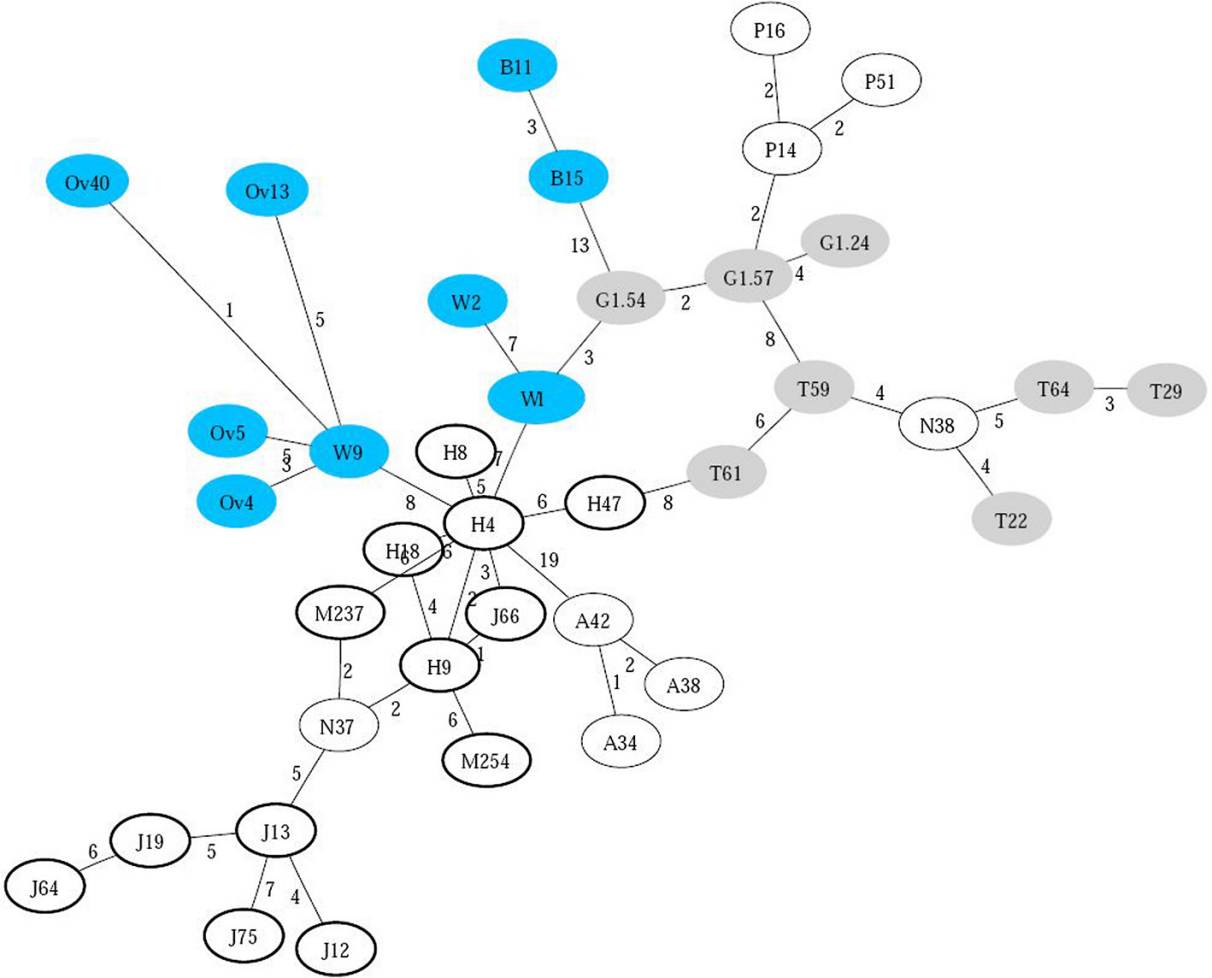
**Ultrametric network of 54 individuals from different populations all over the world.***Δ*=0 and *Δ*=0. The labels represent: H = Han, G = German, T = Turk, J = Japanese, Ov = Ovambo, W = Western Pygmy, A = Australian Aboriginal, P = Papuan, B = San (Bushman), and M= Mongolian. A Rough classification is: J+H+M=East Asian, G+T= Caucasoid, A+P=Australian, B+W+Ov= Africans. Nodes filled with gray represent Caucasoid, the ones filled with blue represent Africans, bold nodes are Asians, and the remaining ones are from Australia.

Figure 3 shows the ultrametric network computed from the first dataset. The labels associated with each node are reported in the figure’s caption, and new nodes are tagged with the letter “N”. The pairwise distances between nodes are reported as attributes of the edges. In Figure 3, the populations are grouped by continent; the nodes filled with gray represent Caucasoid, the ones filled with blue represent Africans, bold nodes are Asians and the remaining ones are from Australia.

**Figure 4:**
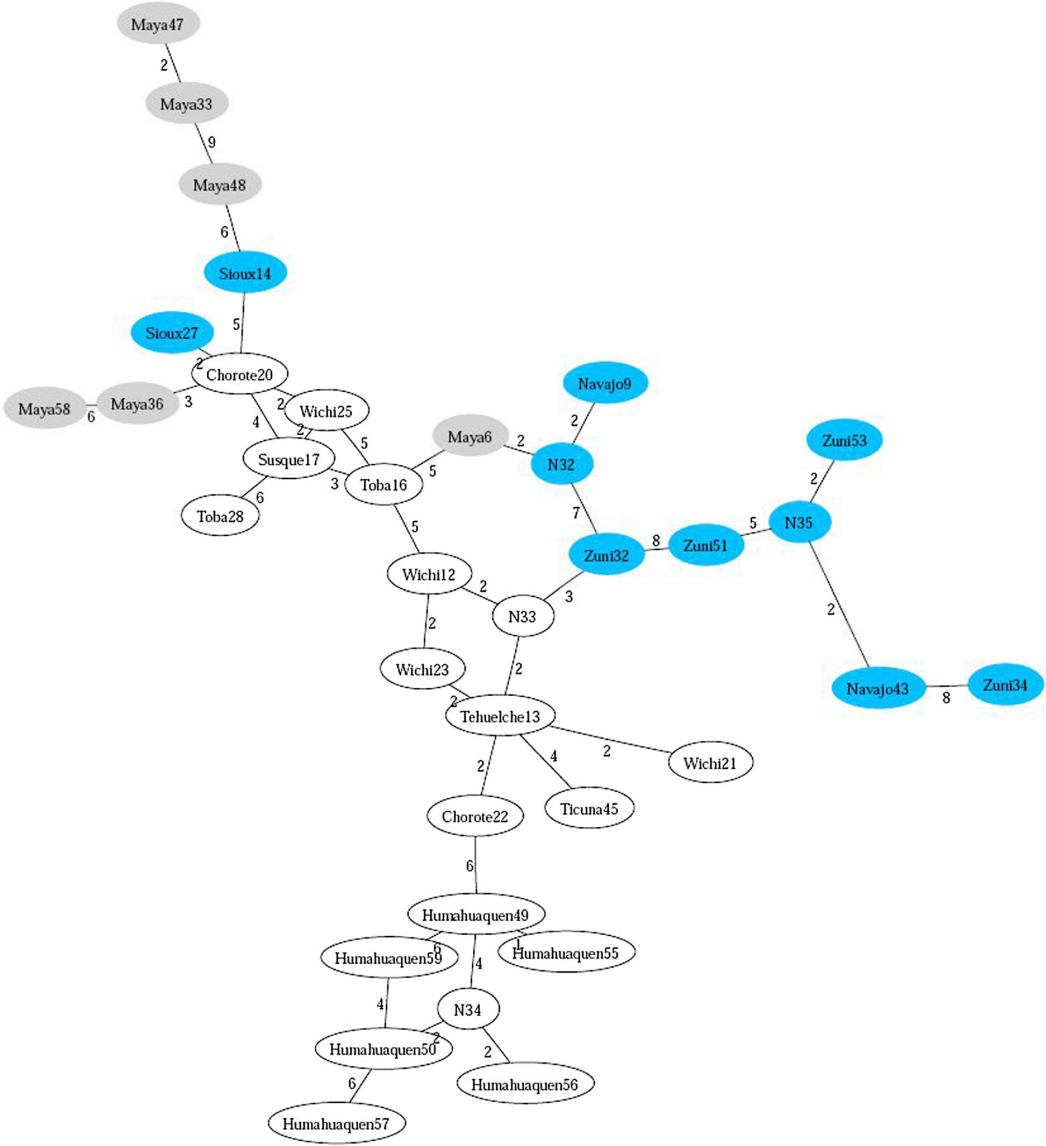
**Ultrametric network of 41 new world natives.** A rough classification is: North America (Navajo, Zuni, Sioux) in blue, Central (Maya) in grey, South (Ticuna, Wichi, Toba, Chorote, Tehuelche), (Susque, Humahuaquen) in white.

We can observe that, in general, all different continents are well separated, and that most of the individuals belonging to the same population are clustered together: Japanese, Han, Turkish, Australian, and so on. Moreover, the known paths of migration support the view that Han are close relatives of Africans and that Japanese evolved from Central Asian populations. Also, Germans appear to be related to Northern Africans and Turks, the latter are also connected with Han, thus supporting the idea that Turks are partially Asians. The only population slightly misplaced are Papuans probably because the STRs examined do not resolve for this population, a problem already observed in [28]. Figure 4 shows the ultrametric network of Native Americans, using the same data as in [29]. This experiment is a particularly difficult test, due to the high level of homoplasy and the small number of STRs available. Nevertheless the network still exposes the structural diversity between North American Natives (blue), Central (gray) and South Americans (white).

**Figure 5:**
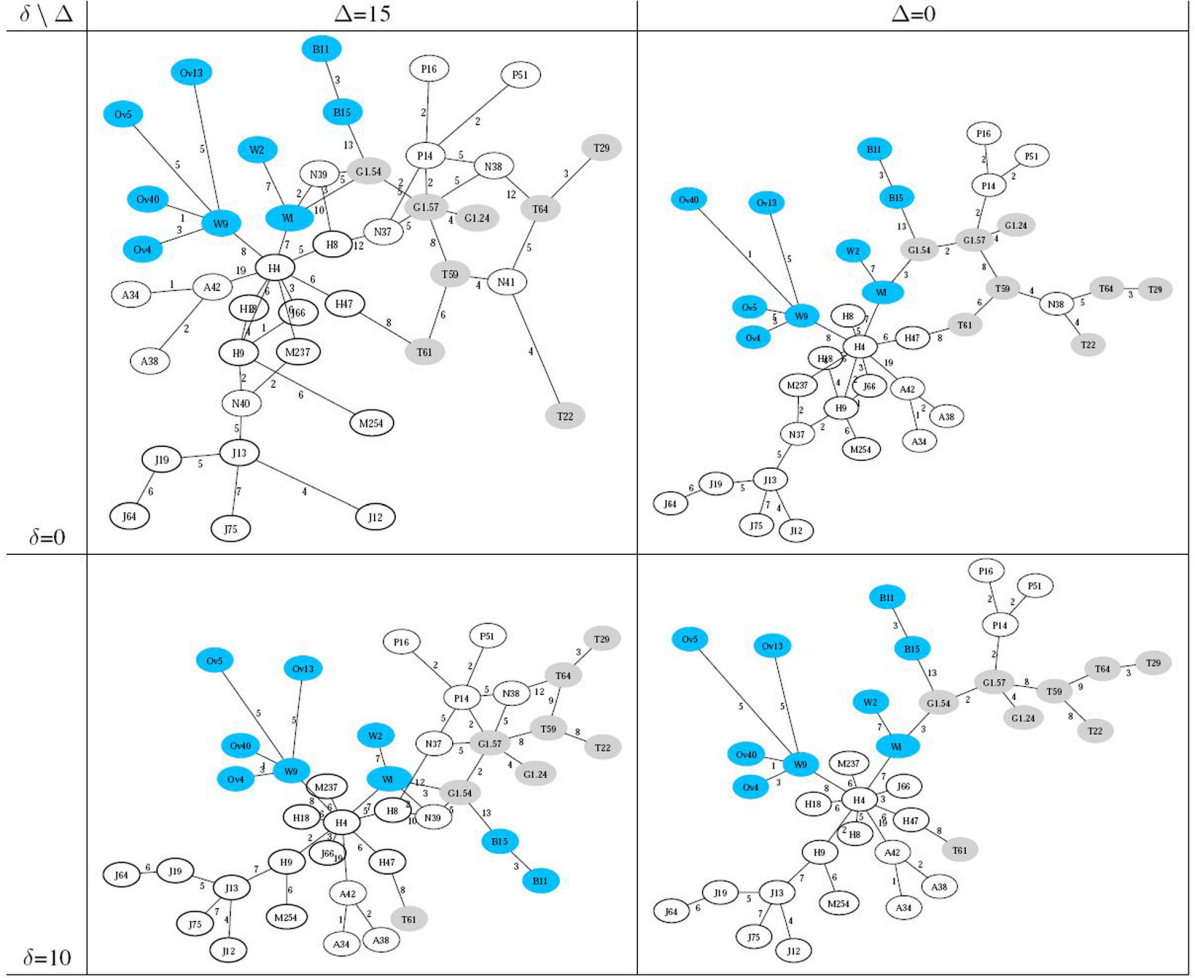
**Ultrametric network of 54 individuals from different populations all over the world (same as in Figure **3) **for a few values of parameters *Δ* and *Δ*.**

As discussed, the connectivity of the inferred network can be fine‐tuned by setting the two control parameters *Δ* and *Δ*. The first one is used to filter out the feeblest edges: with *Δ*=0, all links are selected. The value assigned to the second parameter sets the tolerance within which edges are included in the *Δ*‐ultrametric network. Thus, large values of *Δ* reduce the number of artificial points introduced, large values of *Δ* increase the connectivity of the network. Space limitation prevents a thorough analysis of these variants. As an illustration, Figure 5 displays the networks obtained in correspondence with a few different settings.

## Conclusions

In conclusion, this paper expands the *subdominant ultrametric* perspective by studying ultrametric *networks*. We shown that, for a graph with *n* vertices, the construction of such a network can be carried out by a simple algorithm in optimal time *O*(*n*^2^). This algorithm can be easily adapted to compute relaxed networks, such as *Δ*‐ultrametric networks and to support the introduction of artificial points to reduce the maximum distance between vertices in a pair. Finally, we discussed a few experiments to demonstrate the applicability of subdominant ultrametric networks.

## Competing interests

The authors declare that they have no competing interests.

## Authors’ contributions

All authors contributed equally to this work. All authors read and approved the final manuscript.
